# Anti-Cancer Prodrug Cyclophosphamide Exerts Thrombogenic Effects on Human Venous Endothelial Cells Independent of CYP450 Activation—Relevance to Thrombosis

**DOI:** 10.3390/cells12151965

**Published:** 2023-07-29

**Authors:** Anne Krüger-Genge, Susanne Köhler, Markus Laube, Vanessa Haileka, Sandy Lemm, Karolina Majchrzak, Sarah Kammerer, Christian Schulz, Joachim Storsberg, Jens Pietzsch, Jan-Heiner Küpper, Friedrich Jung

**Affiliations:** 1 Department of Healthcare, Biomaterials and Cosmeceuticals, Fraunhofer Institute for Applied Polymer Research (IAP), 14476 Potsdam, Germany; 2 Institute of Biotechnology, Molecular Cell Biology, Brandenburg University of Technology Cottbus-Senftenberg, 01968 Senftenberg, Germany; 3 Department of Radiopharmaceutical and Chemical Biology, Institute of Radiopharmaceutical Cancer Research, Helmholtz-Zentrum Dresden-Rossendorf, 01328 Dresden, Germany; 4 Faculty of Chemistry and Food Chemistry, School of Science, Technische Universität Dresden, 01069 Dresden, Germany; 5 Brandenburg University of Technology Cottbus-Senftenberg, Fraunhofer Project Group PZ-Syn of the Fraunhofer Institute for Cell Therapy and Immunology, Branch Bioanalytics and Bioprocesses (IZI-BB), 14476 Potsdam, Germany; 6 Faculty of Medicine, Private University in the Principality of Liechtenstein (UFL), 9495 Triesen, Liechtenstein

**Keywords:** cancer, cyclophosphamide, human umbilical vein endothelial cells, HUVEC, liver, cytochrome P450 enzymes (CYP), thrombosis

## Abstract

Cancer patients are at a very high risk of serious thrombotic events, often fatal. The causes discussed include the detachment of thrombogenic particles from tumor cells or the adverse effects of chemotherapeutic agents. Cytostatic agents can either act directly on their targets or, in the case of a prodrug approach, require metabolization for their action. Cyclophosphamide (CPA) is a widely used cytostatic drug that requires prodrug activation by cytochrome P450 enzymes (CYP) in the liver. We hypothesize that CPA could induce thrombosis in one of the following ways: (1) damage to endothelial cells (EC) after intra-endothelial metabolization; or (2) direct damage to EC without prior metabolization. In order to investigate this hypothesis, endothelial cells (HUVEC) were treated with CPA in clinically relevant concentrations for up to 8 days. HUVECs were chosen as a model representing the first place of action after intravenous CPA administration. No expression of CYP2B6, CYP3A4, CYP2C9 and CYP2C19 was found in HUVEC, but a weak expression of CYP2C18 was observed. CPA treatment of HUVEC induced DNA damage and a reduced formation of an EC monolayer and caused an increased release of prostacyclin (PGI2) and thromboxane (TXA) associated with a shift of the PGI2/TXA balance to a prothrombotic state. In an in vivo scenario, such processes would promote the risk of thrombus formation.

## 1. Introduction

Thromboembolic events are known complications of tumor diseases [1,2]. Such events occur about 4 to 7 times more frequently in cancer patients than in the general population [3] and are the second most frequent cause of death in cancer patients [4,5]. Altogether, 15–20% of all cancer patients suffer from thromboembolic complications during their disease [6]. A thrombosis rate of 40.3% has even been demonstrated postmortem [7]. Despite the clear association between malignant disease and thromboembolic events, the molecular and cellular basis of this relationship is still unclear [7,8,9,10,11]. The possible causes have been discussed, including thrombogenic microparticles sheared off from tumor cells or chemotherapeutic drugs, which could directly affect endothelial cell activation and/or perturbation [10]. Thrombotic events might occur due to one or more of the following effects [3,12]:

➢ A reduced antithrombotic function of the endothelial cells in the blood vessel system;
➢ Desquamation of endothelial cells by the dissolution of cell-substrate and/or cell-cell bonds;
➢ By apoptosis/necrosis of endothelial cells (associated with the exposure of the thrombotic subendothelium);
➢ Reduced regeneration capacity of endothelial cells, which finally also could lead to the exposure of the thrombotic subendothelium;
➢ Changes in the glycocalix of the endothelial cells reduce the antithrombotic function.

In cancer therapy, drugs with different modes of action are used. Some of these drugs act directly after their administration without metabolization in the liver, while others only have effects after the active metabolites have been produced by biotransformation (so-called prodrugs [13]). Such a typical prodrug is cyclophosphamide (CPA), a widely used cytostatic drug [14,15]. In order to develop its antitumor activity, CPA is generally thought to require metabolic activation by hepatic cytochrome P450 (CYP) enzymes—a family of enzymes with central importance for the metabolization of endogenous and exogenous compounds. Specifically, the enzymes CYP2B6, CYP3A4/5, CYP2C9, CYP2C18 and CYP2C19 are involved in the metabolization of CPA [16,17,18,19,20,21] to produce the bioactive metabolite phosphoramide mustard (PM), a DNA alkylating agent [22]. Approximately 50% of the drug (unchanged or in the form of metabolites) is excreted via the urine during the first 24 h after administration [23,24,25,26].

CPA is first converted in the liver into 4-hydroxy-cyclophosphamide (4-OH-CPA), which is in steady-state equilibrium with its tautomer aldophosphamide. These unstable metabolites are transported by blood circulation to tumor cells, where they can diffuse freely [26,27]. Intracellularly, aldophosphamide decays to PM by β-elimination of acrolein. As PM should not be able to cross the cell membrane due to its stronger polarity, aldophosphamide is seen as the transport metabolite that enables its penetration into the cell and initiates anti-tumor activity [28]. PM and acrolein are toxic and thus active metabolites [28,29,30]. PM is known to cause inter- and intra-strand DNA crosslinks, leading to DNA double-strand breaks [28,29,30,31,32,33]. If the CPA dose exceeds a certain level, the DNA repair systems can become overloaded, leading to the persistence of DNA double-strand breaks, which then leads to apoptosis or necrosis of the tumor cells [34].

The question arises whether and how cytostatic prodrugs (here cyclophosphamide) can contribute to the development of thromboses and the resulting clinical consequences. Two hypotheses are conceivable for the development of thrombosis due to CPA: (i) venous endothelial cells can metabolize xenobiotics via their own CYP system and thus produce toxic metabolites intracellularly, or (ii) the CPA prodrug could harm endothelial cells without enzymatic activation.

It is well known that endothelial cells, like many other cells, do have a functional NADPH cytochrome P450 oxidoreductase (POR) and CYP system [35,36]. For example, they express CYPs of the CYP3A, CYP2C and CYP2J families [37,38,39,40,41], which produce endogenous mediators such as epoxyeicosatrienoic acids [42]. These CYPs, especially the CYP3A, CYP2B and CYP2C family, are potentially regulated by the nuclear receptor PXR (pregnane X receptor [37,38,39,40,41]). PXR detects foreign substances and activates the expression of enzymes that lead to their metabolization [43]. The expression of PXR mRNA has been demonstrated in human brain capillary endothelial cells and mouse mesenteric arteries [44,45,46,47,48,49]. Furthermore, PXR has been detected in arterial and venous endothelial cells (HUVEC [50,51,52]). Even if endothelial cells use CYP functions for normal physiological processes, it is largely unknown whether this could contribute to any drug-metabolizing activity that is mainly found in hepatocytes. Thus, it is still unclear whether venous endothelial cells can metabolize CPA via CYP enzymes.

Therefore, we investigated (i) whether HUVEC expresses CYP enzymes that can metabolize CPA, (ii) whether active metabolites of CPA can be found in the cell supernatant after treatment, and (iii) whether CPA directly harms HUVEC. To investigate these questions, HUVEC were cultured and investigated for their CPA metabolization capacity, formation of metabolites, the genotoxicity of CPA, cell damage and endothelial cell function. For this purpose, CPA concentrations were used that approximated the plasma levels of patients after intravenous administration.

For the in vitro investigations on the influence of CPA on human venous endothelial cells (HUVEC), drug concentrations were chosen as they are reported to occur in the blood during CPA therapy. After intravenous administration of CPA at a dose of 0.7 g/m^2^ body surface area, the maximum concentration of CPA in plasma immediately after injection was 26.4 ± 3.8 µg/mL (1.94 h after oral ingestion: 22.5 ± 6.9 µg/mL). Elimination times varied considerably (2.9–11.9 h (mean 6.78 h)) so that the endothelium was exposed to CPA for more than 24 h after application. After intravenous high-dose therapy with 60 mg/kg for 2 h in leukemic patients, the mean concentration in plasma after 3 h was 0.257 ± 0.046 mM or 68.12 ± 11.75 µg/mL [53]. For preparing the solution for intravenous infusion, 2 g CPA was dissolved in 100 mL of physiological saline solution. This means that the endothelial cell monolayer of the vein being infused will experience a concentration of 20 mg/mL. In the short term, this is 10 times the dose used in the following preliminary experiments in vitro. Considering these results, CPA was added to the cell culture medium in three concentrations (27 µg/mL, 250 µg/mL, 2500 µg/mL).

## 2. Materials and Methods

All commercial reagents and solvents were analytical reagent grade unless otherwise specified and used without further purification.

HUVECs were used in this study because cyclophosphamide (CPA) is often administered intravenously [24].

### 2.1. Cell Culture

HUVECs (Lonza, Köln, Germany) were cultivated in EBM-2 supplemented with EGM-2 singleQuots kit including Gentamycin-Amphotericin B and 10% fetal bovine serum (EGM-2, Lonza, Köln, Germany [54,55]). Human hepatoblastoma (HepG2) cells (HB-8065; ATCC, Manassas, VA, USA) were cultivated in Dulbecco’s minimal essential medium (DMEM) (Biochrom AG, Berlin, Germany) supplemented with 10% fetal bovine serum (Biochrom AG) and 2 mM L-glutamine (PAA Laboratories GmbH, Pasching, Austria [33]). CYP2C19 and CYP3A4-overexpressing HepG2 clones were additionally cultured with blasticidin (Gibco, Grand Island, NY, USA). Upcyte^®^ hepatocytes (Lot No. 653-03, upcyte technologies GmbH, Hamburg, Germany) were cultivated on 50 µg/mL rat-tail collagen type I (BD Biosciences, San Jose, CA, USA) coated cell culture vessels in Hepatocyte Culture Medium (upcyte technologies GmbH). CHO cells were cultured in Ham’s F-12 (Gibco) with 10% FCS and 2 mM glutamine. All cells were cultivated at 37 °C and 5% CO_2_ in a humidified incubator and routinely passaged by trypsinization with 0.05% (*w*/*v*) Trypsin/0.02% (*w*/*v*) EDTA (Biochrom AG). HUVEC was used for no longer than 4 passages. During the experimental work, cells were checked for mycoplasma infection.

### 2.2. Determination of mRNA Expression Levels of Cytochrome P450 Enzymes (CYPs) and NADPH Cytochrome P450 Oxidoreductase (POR)

Total RNA was extracted from HUVEC, HepG2 and Upcyte^®^ hepatocyte cell pellets (1 × 10^6^ cells) using the innuPREP RNA Mini Kit (Analytik Jena AG, Jena, Germany) according to the manufacturer’s protocol. As enzyme activity of liver cells in vitro declines within hours [37] and further from passage to passage [56,57], previously generated CYP2C19- and CYP3A4-overexpressing HepG2 cells were additionally investigated. CYP-overexpressing HepG2 cells served as positive control (HepG2-2C19 C1 and HepG2-3A4 C9 [33,58]). Chinese hamster ovary (CHO) cells, on the other hand, served as a negative control. In addition, as a positive control, RNA from cryopreserved primary human hepatocytes (CPHH) was used. CPHH-RNA was extracted from Single Freeze Pooled Plateable Cryopreserved Human Hepatocytes (HPCH05+, Pool of 5, Lot No. 1310168, SEKISUI XenoTech, Kansas City, MO, USA) immediately after thawing. Total RNA was treated with DNase I (Thermo Fisher Scientific Inc., Waltham, MA, USA) to remove genomic DNA, and RNA integrity was controlled by 1% agarose gel electrophoresis. Isolated RNA was used for the synthesis of cDNA according to manufacturer’s instruction using oligo(dT)_18_ primer and RevertAid Reverse Transcriptase (Thermo Fisher Scientific). The resulting cDNA was diluted 1:10, and qRT-PCR was performed using primer pairs for different target genes (Table 1; all primers from BioTez, Berlin, Germany). Glyceraldehyde-3-phosphate dehydrogenase (GAPDH) served as reference gene.

Primers, Maxima Probe qPCR Master Mix (Thermo Fisher Scientific), Evagreen (Biotium, Inc., Fremont, CA, USA) and cDNA were mixed accordingly in a 10 µL reaction volume per sample, and the following settings on the iQ^TM^5 Multicolor Real-Timer PCR Detection System (Bio-Rad Laboratories, Inc., Hercules, CA, USA) were used: initial denaturation at 95 °C for 3 min; 50 cycles of 95 °C for 10 s, 62 °C for 10 s and 72 °C for 30 s; final elongation at 72 °C for 30 s. The size of PCR products was controlled by 3% agarose gel electrophoresis. Relative expression levels were calculated from the mean threshold cycle (Ct) values of the target gene minus the mean Ct value of the reference gene GAPDH (delta Ct value).

### 2.3. Ultra-Performance Liquid Chromatography-Tandem Mass Spectrometry (UPLC-MS/MS)

The HEPES-supplemented Krebs-Henseleit-Buffer (pH 7.4, KHB) was freshly prepared using HEPES (5.95 g/L), D-glucose (2.0 g/L), magnesium sulfate (0.141 g/L), potassium dihydrogen phosphate (0.16 g/L), potassium chloride (0.35 g/L), sodium chloride (6.9 g/L), calcium chloride dihydrate (0.373 g/L), and sodium bicarbonate (2.1 g/L) followed by adjustment to pH 7.4 with NaOH and HCl.

Cells were seeded into well plates (6 or 12 wells) in the respective media two days before the incubation experiment. Immediately before incubation with CPA, cells were washed twice with KHB. Then, a solution of 10 mM cyclophosphamide (CPA) in KHB was added (1 mL for 6-well and 500 µL for 12-well) and incubation was carried out for 6 h at 37 °C and 5% CO_2_. For control experiments, incubations were performed in wells without cells using 10 mM cyclophosphamide (CPA) in the indicated buffer solutions. For derivatization of the formed 4-OH-CPA, 30 µL of the supernatant was added to 100 µL semicarbazide hydrochloride (pH 7.4, 0.67 M) and reacted for 60 min at 60 °C. Then, 560 µL acetonitrile was added to each reaction for protein precipitation, and samples were centrifuged at 14,000 rpm at 4 °C for 5 min. Aliquots of the derivatized and precipitated supernatants were withdrawn and subjected to UPLC-MS/MS analysis with system 1 (5–45% gradient). 4-OH-CPA-semicarbazone was monitored in MRM mode at transitions for [M+Na]^+^ 356.096 > 207.057 and for [M+H]^+^ 334.096 > 221.062, as described below. The integrated areas under the curve were normalized to the mean of the signal obtained for HepG2 CYP2C19 in the respective experiment.

UPLC-MS/MS experiments were performed using the following system: UPLC I-Class (binary gradient pump BSM, autosampler FTN, column manager CM, and diode array detector PDAeλ) coupled to a Xevo TQ-S mass spectrometer (Waters, Milford, MA, USA). Aquity UPLC^®^ BEH C18 column (waters, 100 × 2.1 mm, 1.7 µm, 130 Å), eluent: (A): water (Milli-Q grade, Merck Millipore, Burlington, MA, USA), (B): acetonitrile (OPTIMA LC/MS-grade, Fisher Chemical, Hampton, VA, USA); flow rate 0.4 mL/min, 5–45% gradient (t_0_ min 95/5–t_0.5_ min 95/5–t_5.5_ min 55/45–t_6.0_ min 5/95–t_7.0_ min 5/95–t_8.0_ min 95/5–t_8.5_ min 95/5). Mass spectra were obtained by positive electrospray ionization (ESI+) and semi-quantitative analysis was performed using the multiple reaction monitoring (MRM) mode. The MRM settings were optimized using a crude solution of 4-OH-CPA-semicarbazone obtained from the reaction of 4-OOH-CPA in water, which spontaneously forms 4-OH-CPA, with semicarbazide hydrochloride. The Intellistart function (MassLynx 4.1) was used for automated optimization towards the following main ionization and fragmentation parameters: 150 °C source temperature, 500 °C desolvation temperature, 150 °C cone temperature, and 150 L/h cone gas (nitrogen), 1000 L/h desolvation gas (nitrogen) and 0.15 mL/min collision gas (argon) flow. Specific parameters were used as follows: transition 1 for [M+Na]^+^, 356.096 > 207.057, capillary voltage 46 V and collision energy 16 V, 0.0250 s dwell time; transition 2 for [M+H]^+^, 334.096 > 221.062, cone voltage 6 V and collision energy 8 V, 0.0250 s dwell time: The resulting signals for each specific fragmentation were integrated with TargetLynx (version 4.1).

### 2.4. Treatment of HUVEC with Cyclophosphamide

HUVEC were seeded onto glass coverslips in 24-well plates (TPP, Techno Plastic Products AG, Trasadingen, Switzerland) with a cell density of 2 × 10^4^ cells/well. 24 h after seeding, cyclophosphamide monohydrate (Alfa Aesar, Massachusetts, USA) diluted in EGM-2 medium was added in three different concentrations (27 µg/mL, 250 µg/mL, 2500 µg/mL). As (negative) control, cells were cultivated in pure EGM-2 medium without CPA. As a further (positive) control, HUVEC were pro-inflammatory stimulated with 10 ng/mL IL-1ß (as supplement to EGM-2, leading to an activation of the endothelial cells [59]; Invitrogen, Carlsbad, CA, USA). Every second day, the cell culture medium was changed, and all supplements were added freshly. In total, HUVEC were cultured under CPA treatment for eight days to investigate a broad range of cellular reactions, including densities of adherent endothelial cells (number of cells per mm^2^), percentages of viable adherent endothelial cells, metabolic activity, prostacyclin and thromboxane concentration in the cell culture supernatant and the formation of DNA double-strand breaks (γH2AX foci).

#### 2.4.1. Detection of γH2AX Foci by Immunofluorescence

HUVEC were seeded onto glass coverslips and treated with CPA as described above. Eight days after CPA-treatment, the cells were fixed in 4% paraformaldehyde in PBS (Morphisto GmbH, Offenbach am Main, Germany) for 10 min, permeabilized in 0.25% Triton X-100 (Carl Roth GmbH+Co. KG, Karlsruhe, Germany) for 3 min and blocked in 1 × phosphate-buffered saline (PBS, Bio&Sell GmbH, Feucht, Germany) with 3% bovine serum albumin (BSA, Carl Roth GmbH) for 30 min. Thereafter, cells were first incubated with anti-phospho-histone H2AX mouse monoclonal IgG (Merck Millipore) diluted 1:1000 in 1 × PBS/1% BSA for 1 h at room temperature. Then the cells were incubated with polyclonal Cy3-conjugated goat-anti-mouse antibody (Jackson Immuno Research Laboratories, Inc., West Grove, PA, USA) and 0.2 μg/mL DAPI (Carl Roth GmbH) diluted in 1 × PBS/1% BSA for 1 h at room temperature. Immunofluorescence images were obtained with a ZEISS LSM 800 (Carl Zeiss, Jena, Germany). The cell nuclei as well as γH2AX foci were counted using the bioimage analysis software QuPath.

#### 2.4.2. HUVEC Viability

The analysis of cell viability was performed using fluorescein diacetate (FDA, 25 µg/mL Invitrogen, Carlsbad, CA, USA) to stain vital cells in green and propidium iodide (PI, 2 µg/mL, Sigma, Taufkirchen, Germany) to stain dead cells in red. Subsequently, four pictures per sample were taken at 10-fold primary magnification (LSM 510META, Zeiss, Oberkochen, Germany). HUVEC were stained 2, 6 and 9 days after seeding.

#### 2.4.3. Measurement of Metabolic Activity

The mitochondrial activity was measured using the MTS cell Titer 96^®^ Aqueous Non-radioactive Cell Proliferation Assay (MTS assay, Promega, Mannheim, Germany) according to the manufacturer’s instructions.

#### 2.4.4. Analysis of Eicosanoids

Prostacyclin (PGI2) and Thromboxane A2 (TXA2) secretion from adherent HUVEC with and without CPA treatment was quantified 2, 6 and 8 days after CPA addition by using a competitive enzyme-linked immunosorbent assay (ELISA). The concentration of PGI2 was measured in the supernatant of HUVEC using the 6-keto Prostaglandin F_1α_ EIA Kit (Cayman Chemicals, Hamburg, Germany), TXA2 by using the ELISA Kit for Thromboxane A2 (Cloude-Clone Corp., Houston, TX, USA). All analyses were performed according to the manufacturer’s instructions. Eicosanoid concentrations in pg/mL per cell were determined by calculating the values obtained in relation to the number of adherent cells.

### 2.5. Statistics

Data are reported as the arithmetic mean ± standard deviation (SD) for continuous variables. For two-sample problems, a two-tailed *t*-test for paired samples was used, and in the case of three-sample problems, one-factorial ANOVA with *post-hoc* analyses (Tukey test) was used. A *p* value of less than 0.05 was considered significant.

## 3. Results

### 3.1. Lack of Expression of CPA Relevant CYPs in Venous Endothelial Cells

To investigate the potential of HUVEC to metabolize CPA, the expression of CYP2B6, CYP3A4, CYP2C9, CYP2C18 and CYP2C19 and their electron donor protein, NADPH cytochrome P450 oxidoreductase (POR [60]), as CPA metabolizing enzymes, were analyzed in HUVEC. Human hepatoblastoma cells (HepG2), upcyte^®^ hepatocytes and cryopreserved primary human hepatocytes (CPHH) were used as positive controls (liver cells) with known expression of CYPs [61].

**Figure 1:**
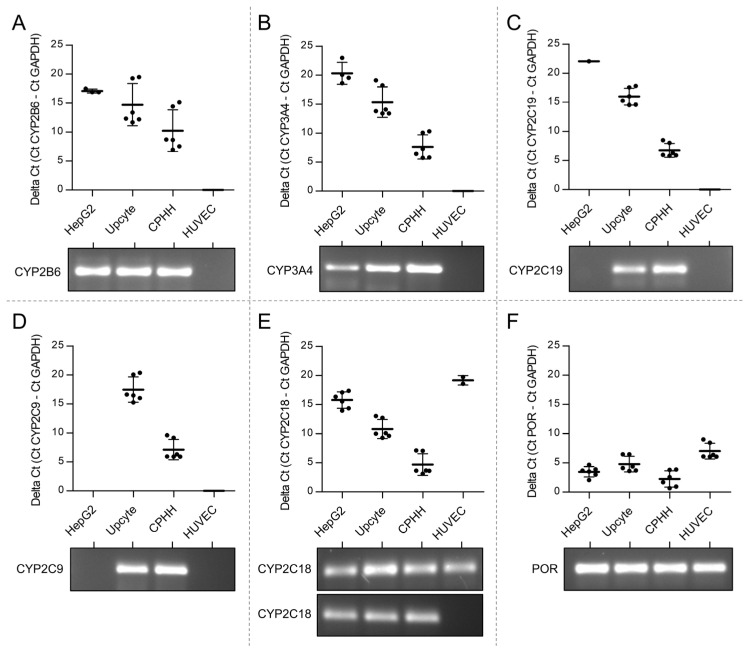
Expression of CYP and POR mRNA in HUVEC. Levels of CYP2B6 (**A**), CYP3A4 (**B**), CYP2C19 (**C**), CYP2C9 (**D**), CYP2C18 (**E**) and POR (**F**) mRNA in different cells were measured using RT-PCR. Upcyte^®^ hepatocytes and cryopreserved primary human hepatocytes (CPHH) were used as positive controls. Relative expression levels of the respective genes are graphically represented by calculating the delta Ct value, representing the difference in the Ct values of the target and the reference gene GAPDH. Results are represented in a scatter blot showing mean values ± SD of three independent experiments. Directly below, a representative agarose gel after electrophoresis of the PCR end products is shown.

The data revealed that HUVEC is not equipped with CYP2B6, CYP3A4, CYP2C9 and CYP2C19 transcripts (Figure 1). Most of the PCR amplicons were below the detection limit. Only the PCR amplicon of CYP2C18 could be weakly detected in two out of six analyses from three independent experiments (Figure 1E). Additionally, POR could be detected in HUVEC (Figure 1F).

### 3.2. Metabolization of CPA in Endothelial Cells

Despite the lack of relevant CYP expression in HUVEC, we wanted to investigate whether there is any formation of the primary CPA metabolite. For this, the occurrence of 4-OH-CPA in the supernatant of HUVEC in comparison to other cells or the diluent buffer (KHB) without cells was analyzed. As CYP enzyme activities of in vitro cultured primary hepatocytes decline within hours and further from passage to passage [56,57,62], previously generated CYP2C19- and CYP3A4-overexpressing HepG2 cells (HepG2-2C19 C1 and HepG2-3A4 C9) were additionally investigated as positive controls [33,58]. Chinese hamster ovary (CHO) cells, on the other hand, served as a negative control.

**Figure 2:**
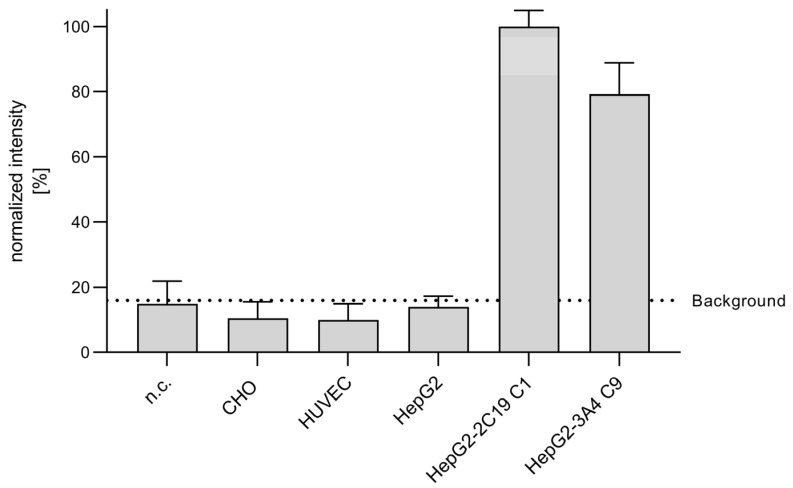
Metabolization of CPA in different cell cultures. CHO, HUVEC, HepG2 and CYP-overexpressing HepG2 cell clones (HepG2-2C19 C1, HepG2-3A4 C9) were exposed to 2500 µg/mL CPA for 6 h. Culture supernatants were used for derivatization of 4-OH-CPA with semicarbazide hydrochloride and subjected to UPLC-MS/MS analysis as described in materials and methods section. 4-OH-CPA-semicarbazone was monitored in MRM mode at transitions for [M+Na]^+^ 356.096 > 207.057. Data represent the mean ± SD of two independent experiments, each performed in duplicate. The intensity of each independent experiment was normalized to the respective mean of the HepG2-2C19 C1 signal. The background (14.9%, 57,575 counts per msec) represents the mean of the control incubations without cells; n.c. (no cells).

Figure 2 shows the normalized intensity of the metabolite 4-OH-CPA for different cell cultures.

In line with the results of the CYP mRNA measurements (Figure 1), above-control levels of 4-OH-CPA could only be detected in CYP-overexpressing cells (HepG2-2C19 C1, HepG2-3A4 C9) but not in HUVEC, CHO or HepG2 cells, a clear hint that HUVEC were not able to metabolize CPA (Figure 2). In contrast, it has been shown that the enzyme activity of CYP-overexpressing HepG2 cells, such as CYP2C19 and 3A4, even approached the physiological value of human hepatocytes within their natural tissue environment [33].

### 3.3. Genotoxicity of CPA in HUVEC

**Figure 3:**
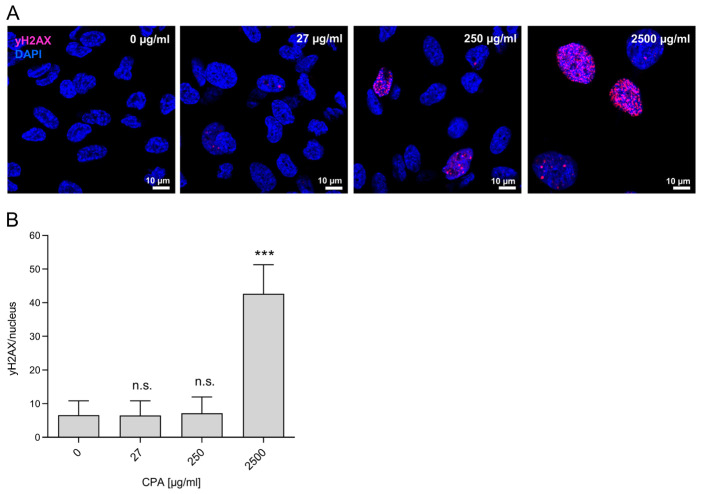
Genotoxicity of CPA in HUVEC. (**A**) Representative immunofluorescence images of HUVEC treated with increasing CPA concentrations for eight days. Images were obtained with a ZEISS LSM 800 (scale bar: 10 µm). (**B**) For quantification, γH2AX foci per nucleus were counted from three independent experiments (mean ± SD is presented); *** *p* < 0.001; n.s.: not significant tested versus dosage 0 µg/mL).

To investigate a possible genotoxic effect of CPA in HUVEC, the formation of γH2AX foci [63,64], a well-known indicator for the formation of DNA double-strand breaks, was investigated [29,65]. The induction of H2AX phosphorylation leading to the formation of γH2AX foci in the cell nuclei was measured in HUVEC after 8 days of exposure to different CPA concentrations (Figure 3). The 8-day incubation of HUVEC with 250 µg/mL CPA resulted in a slight but non-significant increase in γH2AX foci in nuclei (Figure 3A). A further serious increase was found at the highest concentration of 2500 µg/mL (Figure 3B), which was 42.7 ± 8.7 foci/nucleus, 6.5 times higher than for the controls without CPA supplementation (6.6 ± 4.3; *p* < 0.001).

This indicates that CPA can induce DNA damage with known deleterious consequences, especially—if unrepaired—apoptosis or necrosis [66].

### 3.4. Influence of CPA on HUVEC Viability and Function

To investigate the biological effects of CPA on HUVEC, cells were seeded, and 24 h later, CPA was added in three concentrations (27 µg/mL, 250 µg/mL, 2500 µg/mL). Two, six and nine days after cell seeding, HUVEC density, viability, metabolic activity and cell function were investigated.

#### 3.4.1. Decrease in HUVEC Density upon CPA Exposure

**Figure 4:**
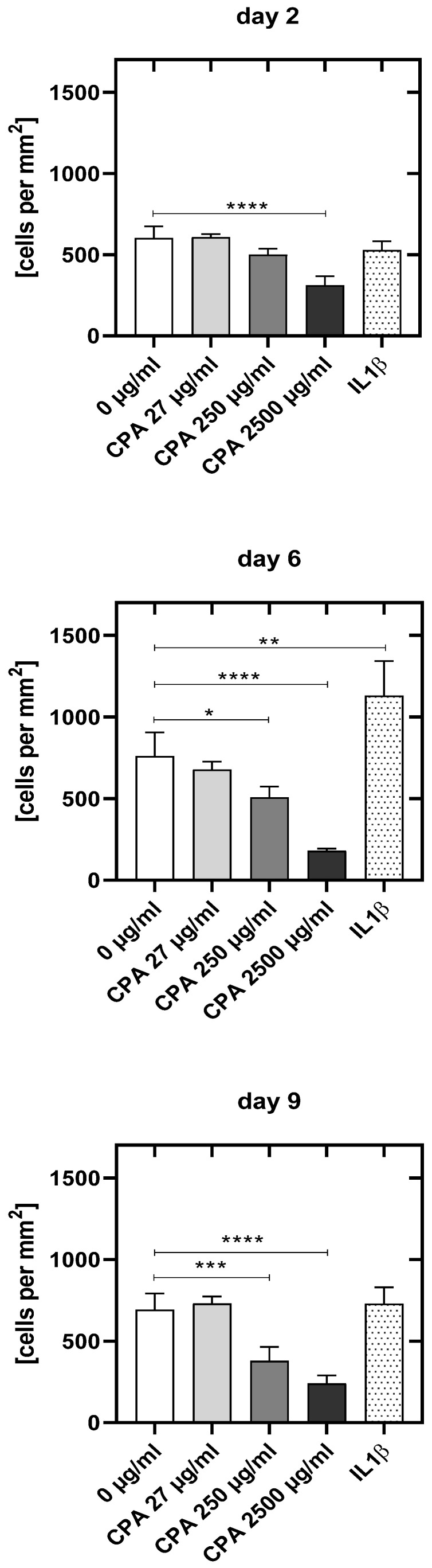
Endothelial cell density (HUVEC/mm^2^) at 2, 6 and 9 days after seeding of cells for the untreated control culture (0 µg/mL), the cell cultures treated with different CPA concentrations (27 µg/mL, 250 µg/mL, 2500 µg/mL) and the cell cultures pro-inflammatory activated with IL1ß. Results are presented as mean values ± SD of four independent experiments (*: *p* < 0.05; **: *p* < 0.01; ***: *p* < 0.001; ****: *p* < 0.0001).

With increasing CPA concentrations, there was a clear decrease in the number of adherent viable HUVEC when compared to the values of the untreated control HUVEC (Figure 4).

HUVEC density was significantly reduced already at day 2 after incubation with the highest CPA concentration (−48.3%, *p* < 0.0001) and thereafter for both the middle and highest concentrations (day 6: 250 µg/mL: 33.1%, *p* < 0.05; 2500 µg/mL: 76.3%, *p* < 0.0001 and day 9: 250 µg/mL: −45.3%, *p* < 0.001; 2500 µg/mL: −65.1%, *p* < 0.0001). The lowest concentration of 27 µg/mL did not decrease HUVEC density within the course of the experiment.

#### 3.4.2. Adherent HUVEC with No Visible Decrease in Viability

**Figure 5:**
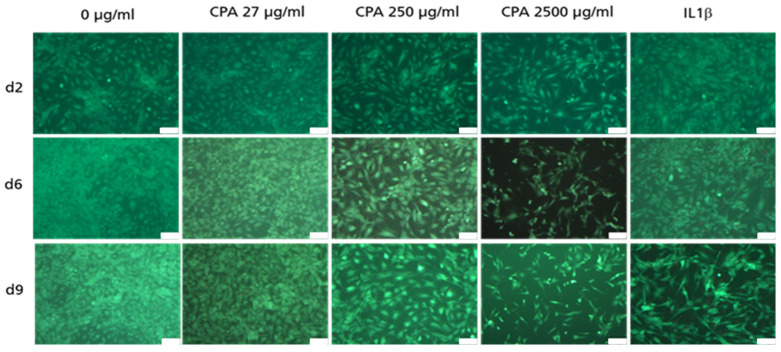
Representative images of FDA (HUVEC in green) or PI (HUVEC in red) stained HUVEC in 10× primary magnification. 2, 6 and 9 days after seeding of the cells for the untreated control culture (0 µg/mL), and after the addition of the different CPA concentrations to cell cultures (27 µg/mL, 250 µg/mL, 2500 µg/mL) and the cell cultures pro-inflammatory activated with IL1ß. Images were obtained using Leica DMI 3000B. Scale bar: 100 µm.

Figure 5 shows adherent HUVEC after staining with FDA/PI. No or nearly no dead HUVEC (in red) were identifiable. This suggests that the viability of the adherent HUVEC was not affected by CPA compared to untreated control cultures (Figure 5).

#### 3.4.3. CPA Concentration-Dependent Decrease in Metabolic Activity in HUVEC

**Figure 6:**
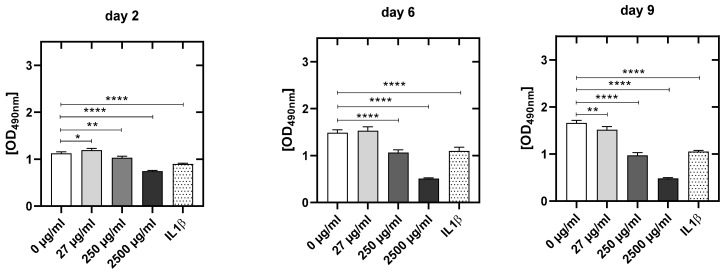
Metabolic activity analysis by MTS assay of HUVEC at 2, 6 and 9 days after seeding of the cells for the untreated control culture (0 µg/mL), and after addition of the different CPA concentrations to cell cultures (27 µg/mL, 250 µg/mL, 2500 µg/mL) and the cell cultures pro-inflammatory activated with IL1ß. Results are presented as mean values ± SD of four independent experiments (*: *p* < 0.05; **: *p* < 0.01; ****: *p* < 0.0001).

CPA reduced the metabolic activity of HUVEC significantly and dose-dependently compared to the values of the untreated control HUVEC (Figure 6). The metabolic activity was significantly reduced at day 2 (250: −8.4%, *p* < 0,01; 2500: −33.7%, *p* < 0.001), at day 6 (250 µg/mL: −29.3%, *p* < 0.001; 2500 µg/mL: −66.7%; *p* = 0.001) as well as at day 9 (27 µg/mL: −10.9%, *p* < 0.01; 250 µg/mL: −42.9%, *p* < 0.05; 2500 µg/mL: −71.8%; *p* = 0.001) (Figure 6).

#### 3.4.4. CPA Affects the Venous Endothelial Cell Function

**Figure 7:**
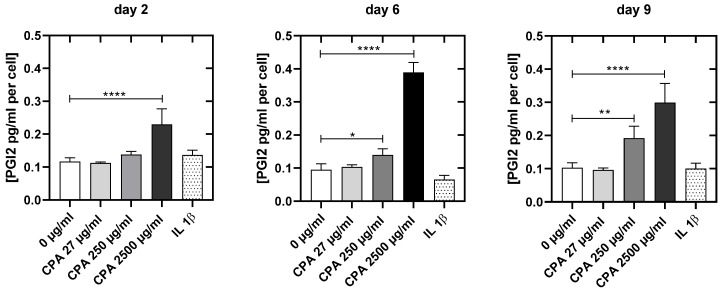
Prostacyclin concentrations (pg/mL per cell) at 2, 6 and 9 days after resettlement of the cells for the untreated control culture (0 µg/mL), the cell cultures treated with different CPA concentrations (27 µg/mL, 250 µg/mL, 2500 mg/mL) and the cell cultures pro-inflammatory activated with IL1ß. Results are presented as mean values ± SD of four independent experiments (*: *p* < 0.05; **: *p* < 0.01; ****: *p* < 0.0001).

With increasing CPA concentrations, the release of prostacyclin (PGI2) increased significantly (Figure 7). While the lowest CPA concentration did not affect the prostacyclin release at any time point, PGI_2_ increased significantly at day 6 (250 µg/mL: +40.0%, *p* < 0.05; 2500 µg/mL: +290%; *p* = 0.001) and especially at day 9 (250 µg/mL: +65.1%, *p* < 0.05; 2500 µg/mL: +157.3%; *p* = 0.001) after the addition of medium and high CPA concentrations.

**Figure 8:**
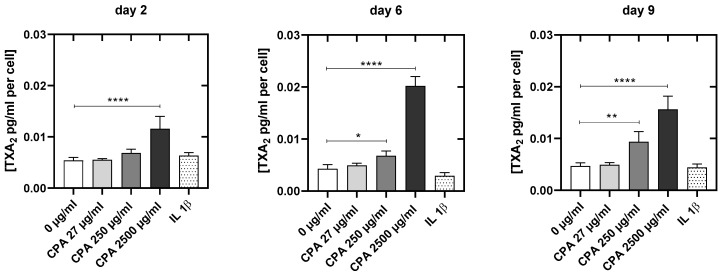
Thromboxane concentrations (pg/mL per cell) at 2, 6 and 9 days after resettlement of the cells for the untreated control culture (0 µg/mL CPA), the cell cultures treated with different CPA concentrations (27 µg/mL, 250 µg/mL, 2500 mg/mL) and the cell cultures pro-inflammatory activated with IL1ß. Results are presented as mean values ± SD of four independent experiments (*: *p* < 0.05; **: *p* < 0.01; ****: *p* < 0.0001).

Next, we tested the effects of CPA on thromboxane production. At day 2, only the highest CPA supplementation led to a significant increase in the thromboxane release into the HUVEC supernatant (2500 µg/mL: +140%, *p* < 0.001). Incubation with the middle and highest concentration for 6 days (250 µg/mL: +75%, *p* < 0.05; 2500 µg/mL: +400%; *p* = 0.001), as well as 9 days (250 µg/mL: +100%, *p* < 0.01; 2500 µg/mL: +300%, *p* = 0.001), resulted in significantly elevated thromboxane levels (Figure 8).

The present study clearly shows that CPA without any detectable enzymatic activation can induce functional impairment in cultivated HUVEC.

## 4. Discussion

The harmful effects of CPA on endothelial cells—as shown in our previous in vitro pilot study [67]—raise the important question of whether CPA injures human venous endothelial cells via an endogenous expression of CPA-metabolizing CYP enzymes analogous to those of hepatocytes. Alternatively, CPA could affect endothelial cell function without prior enzymatic activation. However, this would contradict the widely accepted concept that CPA is a prodrug requiring exclusively hepatic activation by CYP enzymes or would now require a completely different consideration of any side effects, particularly in the vascular system. The present study strongly indicates that human vein endothelial cells are not capable of enzymatically converting CPA into alkylating metabolites (Figure 1 and Figure 2). Here, the expression of the CPA-metabolizing CYP enzymes CYP2B6, CYP3A4, CYP2C9, CYP2C18 and CYP2C19 and their electron donor protein NADPH cytochrome P450 oxidoreductase (POR [60]) as CPA-metabolizing enzymes were analyzed in HUVEC.

**Figure 9:**
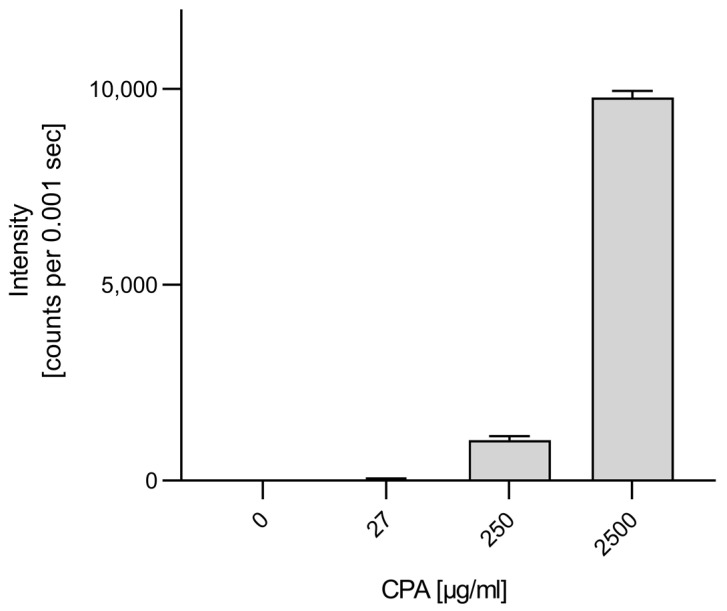
Intensity of 4-OH-CPA in Krebs–Henseleit buffer supplemented with CPA without cells as detected by LC-MS/MS after derivatization to the semicarbazone.

In the first part of the study, various liver cell models (human hepatoblastoma cell line HepG2, upcyte^®^ hepatocytes and cryopreserved primary human hepatocytes, CPHH) were used as positive controls with known expression of CYPs and POR [33,61,68]. Our data reveal that HUVEC does not express the CPA-relevant enzymes CYP2B6, CYP3A4, CYP2C9 and CYP2C19. Although we see a weak band in the agarose gel in two out of six analyses from three independent experiments for CYP2C18, the underlying Ct values were too high to expect an above-background enzyme activity (Figure 1E). Since enzymes only increase the velocity of a product-substrate conversion, it is conceivable that detectable amounts of initial CPA metabolite 4-OH-CPA can also be formed non-enzymatically within the observed time frame. Indeed, Figure 9 shows, that some 4-OH-CPA could also be detected in CPA-supplemented Krebs–Henseleit buffer (without cells), depending on the CPA concentration.

Unfortunately, whether 4-OH-CPA detected in the controls was spontaneously formed from CPA in a non-enzymatic reaction or whether it was already present as an impurity in the commercial CPA batch cannot be assessed from the data collected here since derivatization is required for sensitive detection of 4-OH-CPA. However, 4-OH-CPA has not been described as a major impurity of CPA. Since significant changes in HUVEC function were detected even at the lowest concentration of CPA—at which 4-OH-CPA could not be detected at all—it seems more likely that CPA itself can harm HUVEC (see Figure 6). According to a 1996 guideline of the International Conference on Harmonization of Technical Requirements for Registration of Pharmaceuticals for Human Use (ICH), the top concentration of a pharmaceutical drug to be tested in mammalian cell cultures should not exceed 10 mM. More recently, ICH reduced this concentration to 1 mM (to avoid side effects due to increased osmolality). Typically, 60 mg of CPA for an 80 kg patient is infused, theoretically resulting in a plasma concentration of 1.92 mg/mL (without metabolization and homogeneously distributed in the plasma). Prior to infusion, CPA is reconstituted with normal saline to a concentration of 20 mg/mL (77 mM) CPA. However, circulation of the drug leads to constant dilution and increasingly uniform distribution in about 2.5 L of blood plasma, resulting in a concentration of 68 µg/mL after three hours. To mirror these concentrations in the blood, we chose three concentrations of CPA in the culture medium; a very low concentration of 27 µg/mL, a middle concentration of 250 µg/mL and a high concentration of 2500 µg/mL.

CPA also decays spontaneously in plasma. This spontaneous decomposition produces a degradation product with a toxic effect (nor-nitrogen mustard). However, at a pH of 7.4—as it is present in vivo and in our HUVEC culture—it has no alkylating effect [69]. Thus, a toxic influence on HUVEC by nor-nitrogen mustard can be excluded. The results are thus very likely attributable to CPA itself.

Though no alkylating metabolites above the values of negative controls were found, double-strand breaks could be detected in a dose-dependent manner. Eight days of incubation of HUVEC with CPA led to an increased induction of γH2AX foci. Adding CPA to the HUVEC culture resulted in a significant increase in DNA double-strand breaks at a concentration of 2500 µg/mL (6.6-fold compared to the controls without CPA), while already at 250 µg/mL γH2AX foci in some HUVEC were found. This suggests that CPA itself—without enzymatic conversion into toxic metabolites—can induce DNA damage with known deleterious consequences if unrepaired (Figure 3, [66]). Alternatively, these γH2AX foci were produced by the very small amounts of CPA metabolites formed without enzymatic conversion. In either case, the effects observed strongly argue against the classical prodrug concept of CPA requiring CYP activation. DNA double-strand breaks as consequence of cross-linking of the DNA would induce apoptosis of HUVEC. Apoptotic bodies from endothelial cells could contribute to thrombotic processes due to their procoagulant surface and the expression of highly procoagulant proteins, such as tissue factor (TF) [70], a key activator of the coagulation cascade. The extracellular domain of TF binds and activates FVII, triggering hemostasis, and its aberrant activation causes thrombosis [71]. Besides, endothelial cell activation is also associated with relevant changes in the synthesis of platelet-activating factor (PAF), the release of von Willebrand factor, and the increased production of TF in a paracrine pathway [72,73]. In addition, activated endothelium-induced TF on the cell surface promotes thrombin formation, which might also intensify thrombus formation [74]. In parallel with the occurrence of the γH2AX foci, CPA led, with increasing concentrations, to a decrease in the density of the endothelial cell monolayer compared to the untreated control at day 2 (Figure 4). Double-strand breaks seem to induce HUVEC dysfunction, which might be associated with the detachment of HUVEC and the described decrease in adherent HUVEC after CPA supplementation. In contrast to cultures without CPA, a former study from our group revealed that HUVEC treated with CPA were elongated and showed increased migratory activity [75]. Instead of a confluent HUVEC monolayer as in the control cultures, only the first islets of established HUVEC were visible. Here, we found that CPA led to an activation of those endothelial cells, which was, however, less pronounced than in the cells that had been stimulated with IL1β, a pro-inflammatory cytokine that, among others, converts these in an activated state [76]. However, the viability of the adherent HUVEC was not affected by CPA compared to untreated control cultures. This is well in line with our former study, in which no effect of CPA on HUVEC viability at markedly higher dosages was reported [67].

Supplementation of the HUVEC culture medium with CPA induced a certain dysfunction in HUVEC, a state that was described as perturbation of endothelial cells [67,74,77,78,79,80]. Perturbation of endothelial cells is typically characterized by a significant increase in prostacyclin [81]. Within our study, we observed HUVEC perturbation starting at a concentration of 250 µg/mL CPA, which further increased with increasing drug concentration (Figure 7). Perturbation of HUVEC is shown to induce cyclooxygenase-2, which is instrumental in the increase in prostacyclin generation and release from endothelial cells [79]. The release of prostacyclin is reported to be associated with changes in cell-cell-/cell-substrate-binding [82], coincident with the activation of the microfilament system in HUVEC [83]. In line with these findings, Schnittler et al. described the dissociation of the marginal filament band, a strong induction of stress fibers in central parts of the cells coinciding with progressive inter-endothelial fenestrations [81]. This was associated with the formation of intracellular fenestrations and desquamation of venous endothelial cells. Such pathological aberrations of the venous endothelium could already be shown in vivo [84,85], corroborated by platelet adherence and thrombus formation [86].

At the same time, the thromboxane (TXA) concentrations, a prothrombotic marker [87], in the HUVEC supernatants supplemented with CPA were always significantly higher (up to 400%) than in the control cultures without CPA (Figure 8). This shifts the PGI2/TXA2 balance that is present under physiological conditions [86] towards a prothrombotic state as TXA2 stimulates the activation of platelets as well as platelet aggregation in vivo and in vitro [87,88]. At day 9, the TXA2 increase after CPA addition was 2.5-fold higher than the PGI2 increase. Though the absolute concentration of PGI2 is higher than that of TXA2 (18.7-fold at day 9), the influence of TXA2 on the platelets outweighs this difference because the influence of thromboxane on platelets is four orders of magnitude more potent than prostacyclin. This prothrombotic eicosanoid pattern strongly underlines the thrombotic potential of CPA.

The decreased number of adherent HUVEC in combination with the increase in thromboxane release is in line with a recent animal study. Very recently, Middleton reported that mice treated with CPA four days before intravenous injection of breast cancer cells had more cancer cells in the lung vasculature at 3 h after cancer cell injection than their control counterparts without CPA [88]. The authors discussed the exposure of the subendothelium with increased accessibility of critical protein domains as a cause of the adhesion of cancer cells. In addition, these are also the preferred sites for thrombus formation. Upon detachment of EC, blood components such as proteins or platelets that adhere to the denuded subendothelium, e.g., collagen fibers or von Willebrand molecules, become activated and adhere.

## 5. Conclusions

This study revealed very consistently that treatment with CPA results in significant impairment of endothelial cell function. These included the detachment of HUVEC, which indicates an exposed subendothelium under in vivo conditions, activated (evident by increased prostacyclin) and damaged HUVEC (by increasing γH2AX foci), and the release of prothrombotic thromboxane, which may lead to the activation, adherence and aggregation of platelets [87]. These changes were most likely induced by CPA itself, as neither metabolites of the prodrug were detected above control levels nor were there significant expressions of CYP enzymes metabolizing the drug. However, possible CYP induction by CPA in long-term cultures cannot be ruled out based on the presented results.

In an in vivo scenario, especially in tumor patients with increased procoagulant conditions [8,89,90], such endothelium-mediated processes would promote the risk of thrombus formation, which was detected in more than 40% of patients in a postmortem study [7]. Potentially, the PGI2/TXA2 ratio should be monitored during CPA therapy to consider anticoagulant therapy if significant shifts occur.

To the best of our knowledge, this is the first report showing that CPA at concentrations lower than those occurring in veins during intravenous therapy can induce a condition intravascularly that predisposes thrombosis formation. These findings, supporting our working hypothesis, point to the clinical relevance of intensively investigating possible thrombotic effects of commonly used cytostatic drugs such as CPA to drastically minimize such adverse events.

## 6. Limitations of the Study

Within this paragraph, we wish to highlight the limitations of the proposed study.

1. The CPA concentrations presented in the manuscript were selected in order to cover a large dose range. Here, the bolus administration after the injection should be simulated, in which even much higher intravascular CPA concentrations are present for a short time. However, since we are working in a static in vitro model, it is obvious that there is a significantly higher concentration of CPA in the cell culture than under dynamic conditions in vivo. Thus, the results obtained were also carefully and cautiously discussed and considered from these points of view. Naturally, continuous CPA metabolism cannot be simulated in vitro. This would require the time- and concentration-dependent addition of the active metabolites in vitro or investigations in animal models or in patients.
2. The working group is aware that the selected time intervals are sometimes very long. The time points were selected to ensure that damage to the endothelium could also be recognized after multiple administrations. At the same time, the group wanted to enable the induction of CYPs in the cells through multiple administrations.
3. The working group is aware that in vitro conditions are not comparable with in vivo conditions. Thus, the results obtained here can only allow a first assessment. However, there are clear and significant changes in endothelial cell function toward a prothrombotic state even at the mean CPA concentration.

## Figures and Tables

**Table 1:** Primer sequences for RT-PCR.

Target	Primer 5′-3′	Sequence
**CYP2B6**	Forward	CTCTCCATGACCCACACTAC
	Reverse	TGTTGGGGGTATTTTGCCCA
**CYP3A4**	Forward	GTGGGGCTTTTATGATGGTCA
	Reverse	GCCTCAGATTTCTCACCAACACA
**CYP2C19**	Forward	TTGACCCTCGTCACTTTCTG
	Reverse	GTTGTGTCAAGGTCCTTTGG
**CYP2C9**	Forward	CCAACCCAGAGATGTTTGAC
	Reverse	GAATGAAGCACAGCTGGTAG
**CYP2C18**	Forward	GAGACAACGAGCACCACTCTGA
	Reverse	ACCACAGCATCTGTGTAGGGCA
**POR**	Forward	AAGGCGGTGCCCACATCTAC
	Reverse	TAGCGGCCCTTGGTCATCAG
**GAPDH**	Forward	TGCACCACCAACTGCTTAGC
	Reverse	GGCATGGACTGTGGTCATGAG

## Data Availability

The datasets used in the current study are available from the corresponding authors upon reasonable request.

## Abbreviations

CPA: Cyclophosphamide; CYP: cytochrome P450 enzymes; HUVEC: human umbilical vein endothelial cells; PGI2: prostacyclin; TXA: thromboxane; 4-OH-CPA: 4-hydroxycyclophosphamide; PM: phosphoramide mustard; POR: NADPH cytochrome P450 oxidoreductase; PXR: pregnane X receptor; HepG2: human hepatoblastoma cells; CPHH: primary human hepatocytes; CHO: chines hamster ovary; UPLC-MS/MS: ultra-performance liquid chromatography-tandem mass spectrometry.
